# *RAD51* and Breast Cancer Susceptibility: No Evidence for Rare Variant Association in the Breast Cancer Family Registry Study

**DOI:** 10.1371/journal.pone.0052374

**Published:** 2012-12-27

**Authors:** Florence Le Calvez-Kelm, Javier Oliver, Francesca Damiola, Nathalie Forey, Nivonirina Robinot, Geoffroy Durand, Catherine Voegele, Maxime P. Vallée, Graham Byrnes, Breast Cancer Family Registry, John L. Hopper, Melissa C. Southey, Irene L. Andrulis, Esther M. John, Sean V. Tavtigian, Fabienne Lesueur

**Affiliations:** 1 Genetic Cancer Susceptibility Group, International Agency for Research on Cancer, Lyon, France; 2 Biostatistics Group, International Agency for Research on Cancer, Lyon, France; 3 Center for Molecular, Environmental, Genetic and Analytical Epidemiology, School of Population Health, EGA The University of Melbourne, Victoria, Australia; 4 Genetic Epidemiology Laboratory, The University of Melbourne, Victoria, Australia; 5 Department of Molecular Genetics, Samuel Lunenfeld Research Institute, Mount Sinai Hospital, Toronto, Ontario, Canada; 6 Cancer Prevention Institute of California, Fremont, California, United States of America; 7 Stanford University School of Medicine and Stanford Cancer Institute, Stanford, California, United States of America; 8 Department of Oncological Sciences, Huntsman Cancer Institute, University of Utah School of Medicine, Salt Lake City, Utah, United States of America; IFOM, Fondazione Istituto FIRC di Oncologia Molecolare, Italy

## Abstract

**Background:**

Although inherited breast cancer has been associated with germline mutations in genes that are functionally involved in the DNA homologous recombination repair (HRR) pathway, including *BRCA1*, *BRCA2*, *TP53*, *ATM*, *BRIP1*, *CHEK2* and *PALB2,* about 70% of breast cancer heritability remains unexplained. Because of their critical functions in maintaining genome integrity and already well-established associations with breast cancer susceptibility, it is likely that additional genes involved in the HRR pathway harbor sequence variants associated with increased risk of breast cancer. *RAD51* plays a central biological function in DNA repair and despite the fact that rare, likely dysfunctional variants in three of its five paralogs, *RAD51C, RAD51D,* and *XRCC2,* have been associated with breast and/or ovarian cancer risk, no population-based case-control mutation screening data are available for the *RAD51* gene. We thus postulated that *RAD51* could harbor rare germline mutations that confer increased risk of breast cancer.

**Methodology/Principal Findings:**

We screened the coding exons and proximal splice junction regions of the gene for germline sequence variation in 1,330 early-onset breast cancer cases and 1,123 controls from the Breast Cancer Family Registry, using the same population-based sampling and analytical strategy that we developed for assessment of rare sequence variants in *ATM* and *CHEK2.* In total, 12 distinct very rare or private variants were characterized in *RAD51*, with 10 cases (0.75%) and 9 controls (0.80%) carrying such a variant. Variants were either likely neutral missense substitutions (3), silent substitutions (4) or non-coding substitutions (5) that were predicted to have little effect on efficiency of the splicing machinery.

**Conclusion:**

Altogether, our data suggest that *RAD51* tolerates so little dysfunctional sequence variation that rare variants in the gene contribute little, if anything, to breast cancer susceptibility.

## Introduction

Reliable DNA double-strand break (DSBs) repair machinery is essential for the maintenance of genome integrity, and dysfunctional proteins involved in detecting DSBs have been shown to lead to malignancy [1]. Inherited breast cancer has been associated with germline mutations in genes functionally involved in the homologous recombination pathway of DSB repair. These include rare highly penetrant mutations in *BRCA1* [2], [3], *BRCA2* [4], and *TP53* [5], and rare intermediate-risk variants in *ATM* [6]-[8], *BRIP1* [9], *CHEK2* [10], [11], *PALB2* [12]-[14], and *XRCC2* [15]. However, despite important progress in understanding genetic determinants of breast cancer, only about 30% of the familial relative risk can be explained by the known breast cancer susceptibility alleles, suggesting that mutations in other biologically relevant genes remain to be identified. Because of the HRR pathway’s critical functions in maintaining genome integrity, it is likely that additional genes involved in HRR harbor rare dysfunctional sequence variants that explain part of the missing heritability of breast cancer.

RAD51 (MIM 179617; NM_002875.4) is the central protein of homologous recombination, playing the critical role of catalyzing the strand transfer between a broken sequence and its undamaged homologue in order to re-synthesize the damaged region [16]. The RAD51 recombinase is also involved in a complex network of cellular damage-sensing and cell cycle checkpoint signaling pathways interacting with p53 [17], BRCA1 [18], BRCA2 [19] and PALB2 [20]. Therefore, dysregulation of RAD51 can lead to impaired HRR and gross re-arrangement aberrations, which are genetic events often observed in cancers. Moreover, dysregulation of RAD51 expression in breast cancer cells has been reported. Some studies have reported concomitant down-regulation of BRCA1 and increase of RAD51 levels in sporadic invasive ductal breast cancer [21], and others reported reduced levels of both proteins in breast tumor cell lines and breast cancer cells [22], leading to considerable speculation about the role of RAD51 in breast tumorigenesis. At the germline level, some studies have shown that the single nucleotide polymorphism (SNP) −135G>C (rs1801320) in the 5′ untranslated region (UTR) of *RAD51* modifies breast cancer risk in *BRCA2* mutation carriers but not in *BRCA1* mutation carriers [23], [24]. These results were later confirmed in a combined analysis of 19 studies [25]. Subsequently, a number of other meta-analyses attempted to evaluate the contribution of the −135G>C SNP to breast cancer susceptibility in the general population [26]-[29], but results are conflicting [30], [31]. To our knowledge, only three studies have investigated the contribution of rarer inherited *RAD51* sequence variants in breast cancer susceptibility [32]-[34]. Rapakko *et al*. screened the entire coding region of the gene in 126 Finnish breast and/or ovarian cancer families (10 of which were proven to carry a *BRCA1* or *BRCA2* mutation), and and Lose *et al*. screened 46 Australian *BRCA1* and *BRCA2*-negative breast cancer families. Both groups reported no disease-related *RAD51* alterations. Kato *et al.* screened *RAD51* for germline mutations in 45 Japanese high-risk breast cancer patients and reported a single missense variant, p.Arg150Gln, which was proposed to be the disease-causing mutation in two unrelated Japanese patients with bilateral breast cancer and with a familial history of the disease. Functional analysis revealed that the p.Arg150Gln mutant exhibited reduced ssDNA- and dsDNA-binding abilities that could contribute to breast tumorigenesis in patients carrying such a mutation [35]. Since previous studies were based on very small numbers of patients, a larger population-based study to investigate the contribution and frequency spectrum of *RAD51* germline variants to breast cancer susceptibility is warranted.

Following a similar *in silico*-driven analysis strategy to that we developed to handle the case-control mutation screening data of *ATM* [7] and *CHEK2* [11], we investigated the distribution of rare coding variants in the Breast Cancer Family Registry (BCFR) population-based series composed of 1,330 early onset breast cancer cases and 1,123 controls. In addition, we examined the controversial −135G>C SNP in this case-control series.

## Results

We screened the entire coding region and flanking exon boundaries of the *RAD51* gene for germline sequence variations in 2,453 BCFR subjects. We identified 3 missense substitutions, 4 silent substitutions and 5 non-coding variants with allele frequencies lower than 1% in the studied populations (Table 1). All 3 missense substitutions (p.Arg150Gln, p.Ala224Gly, p.Met326Val) were assigned to grade C0 (the most benign grade) by Align-GVGD [36], and pArg150Gln and p.Met326Val were predicted to be neutral by SIFT and PolyPhen2.1, which are two other popular *in silico* prediction tools. Of note, the missense substitution p.Ala224Gly observed in one control was predicted to affect protein function by SIFT and predicted as possibly damaging by PolyPhen2.1 (Table 1). There was no difference in the distribution of the missense substitutions in cases versus controls (P_logit_ = 0.88, with race/ethnicity, study center and age included in the regression model as covariates). To further investigate whether any of the sequence variants could alter proper splicing of *RAD51,* we scored all missense substitutions, silent substitutions and intronic variants detected in the vicinity of splice junction consensus sites on splicing with NNSplice [37] and MaxEntScan [38] programs. NNSplice is a splice site detection algorithm based on neural networks, which finds and scores potential splice sites in a given sequence, while MaxEntScan’s algorithm computes the maximum entropy score of a given sequence using human training splice site models. Conjunction of both splice sites algorithms was thus used to evaluate the risk for a sequence variant to disrupt the splicing machinery: either by destroying a wild-type splice site or by creating a *de novo* splice site. Two intronic variants, c.531-31 C>T and c.896+33 G>A have modest potential to create *de novo* acceptors. In the case of c.531-31 C>T, the C allele has a MaxEntScan (MES) acceptor score of 4.98 with a potential splice site located three bp downstream of the variant. The T allele reduces the MES score slightly to 4.26. In contrast, the native acceptor for this exon has a much higher MES score, 11.97. Thus the probability of an effect on splicing is modest. In the case of c.896+33 G>A, the G allele has an MES acceptor score of −1.64 two bp downstream of the sequence variant. The A allele raises that score notably, to 6.31, whereas the native acceptor for this exon has a slightly higher MES score of 7.81. However, the sequence variant is too close to the end of exon 9 to be expected to create a functional acceptor; consequently, the probability of an effect on splicing is again modest. Functional assays however, are required to confirm these predictions. The impact of the c.-1dupA variant is unknown and it would also require functional assays to better characterize its effect on the protein function; however, no material was available for the carriers of these three variants to further investigate their functional role *in vitro*.

**Table 1:** Rare genetic variants of *RAD51* identified in the BCFR.

	Variants	Effect on protein	Align-GVGD*	SIFT*	PolyPhen2.1 (HumDiv)	Cases (*N* = 1,330)	Controls (*N* = 1,123)
*Missense substitutions*							
c.449 G>A		p.Arg150Gln	C0	0.68	Benign	1	1
c.671 C>G		p.Ala224Gly	C0	0.03	Poss. damaging	0	1
c.976 A>G		p.Met326Val	C0	0.09	Benign	1	0
*Silent substitutions*							
c.108 C>T		p.Asn36Asn	–	–	–	0	1
c.414 T>C		p.His138His	–	–	–	0	1
c.645 G>A		p.Arg215Arg	–	–	–	0	1
c.720 C>G		p.Ala240Ala	–	–	–	1	1
*Other types of variants*							
c.-2–19 A>G		NA	–	–	–	2	2
c.-1dupA		Unknown	–	–	–	1	0
c.531-31 C>T		NA	–	–	–	2	0
c.896+5delG		NA	–	–	–	1	1
c.896+33 G>A		NA	–	–	–	1	0

* A protein multiple sequence alignment (PMSA) including 15 sequences from Human to Drosophila (Dmel) was used to obtain scores for Align-GVGD and for SIFT (Median sequence conservation of 4.32 substitutions per position).

NA, not applicable.

Because of the controversial results reported in the literature about the common 5′UTR polymorphism (−135G/C. rs1801320), we also genotyped the BCFR series for this SNP. We observed some heterogeneity in the allele frequencies in the four racial/ethnic groups represented in this BCFR series, and found that the minor allele (C) in European, Latina and subjects of recent African ancestry was the major allele in East Asian population. The observed frequency of the C allele in the four racial/ethnic groups is shown in Table 2. No departure from Hardy-Weinberg equilibrium was observed in either cases or controls. Overall, there was no significant difference in allele frequencies between cases and controls for SNP RAD51 −135G/C when pooling together the different populations. Interestingly, after stratifying by race/ethnicity, we detected a significant under-representation of the minor allele C in the East Asian population (*P* = 0.044). In the genotype analysis, the best fitting model was the log-additive model indicative of a possible protective dose effect of the variant [odds ratio (OR) 0.56, 95% confidence interval (CI) 0.33, 0.95]. Further investigation in larger Asian case-control studies would be warranted to clarify the contribution of this SNP in this population.

**Table 2:** Stratified analyses of the *RAD51* −135G/C SNP on breast cancer risk in the BCFR.

	Number of genotyped subjects	C allele frequency		Log-additive model^b^	
	Cases/Controls	Cases/Controls	Chi^2^ *P*-value^a^	OR [95% CI]	*P*-trend
Combined	1,193/1,019	0.089/0.080	0.42	0.89 [0.71, 1.27]	0.30
By race/ethnicity					
European	763/873	0.061/0.066	0.56	0.95 [0.71, 1.27]	0.71
East Asian	187/69	0.131/0.203	**0.044**	**0.56 [0.33, 0.95]**	**0.033**
Recent Africanancestry	91/32	0.236/0.203	0.59	1.36 [0.69, 2.71]	0.38
Latina	152/45	0.072/0.078	0.86	0.87 [0.33, 2.29]	0.77

a Test for the difference in C allele frequency between cases and controls.

b Results of the logistic regression assuming a log-additive model with study center and age included in the regression model as covariates in the combined analysis, and with race/ethnicity, study center and age as covariates in the stratified analysis.

During the course of the mutation screening, we also identify seven intronic SNPs which are though to be innocuous, as they all fall quite far from the intron-exon boundaries and are unlikely to creat cryptic splicing sites. We found no significant difference in allele frequencies between cases and controls for these non-coding SNPs when pooling together the different populations. In population-specific analysis, we observed a potential association for c.344-36T>G in East Asian population (p = 0.015) and a potential association for c.896+86C>T in the population of recent African ancestry (p = 0.043). However, due to the number of tests that have been performed, results were not significant after correcting for multiple testing. (Table S1).

## Discussion

To our knowledge, this is the largest case-control mutation screening study that investigated whether rare sequence variation within *RAD51* contributes to breast cancer susceptibility. The gene was an interesting candidate because of (i) its central role in recombination and DNA repair, (ii) its physical interaction with the two well known breast cancer susceptibility products BRCA1 and BRCA2 that activate a DNA damage response pathway involving both recombination and double-strand break repair and (iii) the identification in breast and/or ovarian cancer families of germline deleterious mutations in three of its five paralogs, *RAD51C, RAD51D,* and *XRCC2* [15], [39]-[42].

We identified 12 distinct rare *RAD51* variants in 0.75% (10/1.330) of early-onset breast cancer cases and 0.8% (9/1.123) of controls, all but one of them novel. The one substitution that had been reported previously, p.Arg150Gln, had been observed in two unrelated patients with familial breast cancer, one with synchronous bilateral breast cancer and the other with synchronous bilateral multiple breast cancer [32], [33]. While the authors of the p.Arg150Gln work concluded after demonstrating the absence of the mutation in 200 sporadic breast cancers and in 100 colon cancers that the *RAD51* p.Arg150Gln substitution was likely to be the disease-causing mutation, we could not confirm those findings, as we found no difference in occurrence of this mutation between cases and controls. Scores from Align-GVGD and SIFT were both indicative of an innocuous variant. All of the tetrapods in our RAD51 protein multiple sequence alignment (available online at http://agvgd.iarc.fr/alignments) have arginine at this position. However, zebrafish, lancelet, tunicate and fruit fly all have glutamine at this position, leading to an inference that glutamine was the ancestral chordate residue at this position and that glutamine at this position is compatible with functional RAD51 protein. Thus while preliminary functional analysis has reported that the purified RAD51 (pArg150Gln) mutant protein exihibited decresead ssDNA- and dsDNA-binding properties [35], further biochemical and structural analyses would be required to better characterize the impact of the *RAD51* p.Arg150Gln variant on the human protein.

Altogether, it seems that germline mutations in *RAD51* would probably be too damaging to be tolerated. This view is further supported by the observation that disruption of the *RAD51* gene leads to embryonic lethality in mice [43], [44]. Indeed, BRCA2 binds RAD51 through the very well conserved BRCA2 BRC repeats [19], [45]. Pellegrini et al (2002) reported the structure of the complex between the BRC repeat and the RecA-homology domain of RAD51 and concluded that the BRC repeat mimics a motif in RAD51 enabling BRCA2 to control the assembly of the RAD51 nucleoprotein filament, which is essential for strand-pairing reactions during DNA recombination [46]. They also showed that defective binding at a single BRC repeat impairs BRCA2-RAD51 interaction. In a yeast two-hybrid experiment and in vitro binding assays. Wong et al reported that the C-terminus of RAD51 (codons 98-339) is crucial for interaction with the very well conserved BRC repeats of BRCA2, indicating that more than 70% of the amino acid sequence is required for RAD51 to bind BRCA2. which also suggests that a dysfunctional mutation in the C-terminal domain would impair the recombinational repair of double-strand DNA breaks [19]. However, Park *et al.* (2008) identified a *RAD51* splice variant lacking exon 9, called RAD51-delta-ex9. The deduced 280-amino acid protein is identical to full-length RAD51 for the first 259 amino acids, which includes an N-terminal basic motif followed by the Walker A and B ATP-binding motifs, but diverges at its C terminus, suggesting that this variant may have a different binding property from the full-length RAD51 in interaction with BRCA2. In spite of this, the authors reported that the RAD51-delta-ex9 exhibited a DNA-strand exchange activity comparable to that of full-length RAD51, suggesting that alternative pathways involving RAD51 exist for DNA recombination and repair [47]. Moreover, using the crystal structure of full-length archaeal RAD51 and mutation analysis in *Pyrococcus furiosus*, Shin et al (2003) found that the interaction between BRCA2 BRC repeats and RAD51 mimic RAD51 polymerization; they also demonstrated the existence of analogous RAD51 interactions with the RAD51 partners RAD52 and RAD54 [48]. Taken together these results demonstrated that the RAD51 polymerisation motif and associated polymeric interface act as a probable platform that promotes and coordinates homologous recombinational repair pathway progression. Altogether, those studies, in conjunction with our study, seem to corroborate the idea that RAD51 is playing a too central and crucial role in homologous recombination and DSBs repair involving multiple pathways and complexes to tolerate deleterious germline variations and therefore contributes very little, if anything, to breast cancer susceptibility.

*RAD51* has the least cross-species sequence variation (as percent amino acid sequence identity between human and other species represented in the alignments) of the genes that we have subjected to case-control mutation screening (Table 3) [7], [11], [15]. Indeed, the curated protein multiple sequence alignment (PMSA) we built to score the missense substitutions, which includes 15 sequences from Human to Drosophila (Dmel), has a maximum parsimony estimate of only about one substitution per position (Ideally, three subtitutions per position is the criterion for use with Align-GVGD). In parallel, *RAD51* presents the lowest load of rare missense substitutions (substitutions position^−1^ person^−1^) of the genes that we have subjected to case-control mutation screening (3.6×10^−6^ substitutions position^−1^ person^−1^ were observed for *RAD51 versus* 26.0×10^−6^ substitutions position^−1^ person^−1^ for *CHEK2*, 19.4×10^−6^ substitutions position^−1^ person^−1^ for *ATM* and 8.8×10^−6^ substitutions position^−1^ person^−1^ for *XRCC2* in the previous case-control mutation screening studies we conducted in the BCFR series). Hence, while our data do not demonstrate that severely dysfunctional sequence variants in *RAD51* confer increased risk of breast cancer, the data support the hypothesis that, probably due to intense purifying selection, such variants are so rare in the human population that their contribution to the attributable fraction and familial relative risk of breast is very small.

**Table 3:** RAD51 protein multiple sequence alignment characterization.

Sequencesource	Sequence length	Ave. number of substitutions per position	SIFT: median sequence conservation score	Percent amino acid sequence identity in pairwise comparison														
				Hsap	Mmus	Rnor	Ocun	Cfam	Btau	Sscr	Mdom	Ggal	Xlae	Drer	Bflo	Cint	Spur	Dmel
Hsap	339	na	na	1.00														
Mmus	339	nm	4.32	0.99	1.00													
Rnor	339	nm	4.32	0.99	0.99	1.00												
Ocun	339	nm	4.32	0.99	0.99	0.99	1.00											
Cfam	339	nm	4.32	0.99	0.99	0.99	0.99	1.00										
Btau	339	nm	4.32	0.99	0.99	0.99	0.99	1.00	1.00									
Sscr	339	nm	4.32	0.99	0.98	0.98	0.98	0.99	0.99	1.00								
Mdom	339	nm	4.32	0.96	0.97	0.97	0.96	0.96	0.96	0.95	1.00							
Ggal	339	nm	4.32	0.96	0.96	0.96	0.95	0.95	0.96	0.94	0.95	1.00						
Xlae	336	nm	4.32	0.96	0.95	0.95	0.94	0.95	0.95	0.95	0.93	0.94	1.00					
Drer	340	nm	4.32	0.90	0.90	0.89	0.90	0.90	0.90	0.90	0.88	0.88	0.91	1.00				
Bflo	339	nm	4.32	0.85	0.84	0.85	0.84	0.84	0.85	0.84	0.83	0.83	0.85	0.83	1.00			
Cint	338	0.64	4.32	0.82	0.82	0.82	0.81	0.82	0.82	0.82	0.82	0.80	0.80	0.81	0.78	1.00		
Spur	335	0.80	4.32	0.84	0.84	0.84	0.83	0.84	0.84	0.84	0.84	0.84	0.84	0.83	0.82	0.80	1.00	
Dmel	336	1.19	4.32	0.66	0.66	0.66	0.66	0.66	0.66	0.66	0.65	0.66	0.67	0.68	0.64	0.64	0.69	1.00

na Not applicable.

nm Not measured. These measurements were conducted by sequentially removing sequences from the “complete” alignment and then performing the analysis indicated. Thus there was no need to continue after the alignment failed to meet either informativeness criterion.

Species abbreviations: Hsap, Homo sapiens (human); Mmus, Mus musculus (mouse); Rnor, Rattus norvegicus (rat); Ocun, Oryctolagus cuniculus (rabbit); Cfam, Canis familiaris (dog); Btau, Bos taurus (cow); Sscr, Sus scofa (pig); Mdom, Monodelphis domesticus (grey, short tailed opossum); Monodelphis domestica (opossum); Ggal, Gallus gallus (chicken); Xlae, Xenopus laevis (frog); Drer, Danio rerio (zebrafish); Bflo, Branchiostoma floridae (lancelet); Cint, Ciona intestinalis (tunicate); Spur, Strongylocentrotus purpuratus (purple sea urchin); Dmel, Drosophila melanogaster (fruitfly).

Nonetheless, we cannot rule out the possible implication of extremely rare *RAD51* deleterious mutations in some high-risk families with specific phenotypes. Indeed, *RAD51C* and *RAD51D* mutations have been specifically found in breast plus ovarian cancer families. A large case-control mutation screening study focusing on ovarian cancer would also be warranted to investigate the implication of *RAD51* in ovarian cancer susceptibility.

## Materials and Methods

### Ethics Statement

*RAD51* mutation screening and analyses described here were approved by the Ethics committee of the International Agency for Research on Cancer (IARC), the University of Utah Institutional Review Board (IRB), and the local IRBs of the Breast Cancer Family Registry (BCFR) centers from which we received samples. These local IRBs were the Health Sciences Human Ethics Subcommittee of the University of Melbourne, Australia; the Institutional Review Board of the Northern California Cancer Center (now the Cancer Prevention Institute of California); and the Research Ethics Board of Mount Sinai Hospital, Ontario, Canada. All participants gave written informed consent.

### Subjects

Subjects were selected from women ascertained by population-based sampling by the BCFR at three centers (Cancer Care Ontario. the Cancer Prevention Institute of California (formerly the Northern California Cancer Center), and the University of Melbourne) [49]. Subjects were recruited between 1995 and 2005. Selection criteria for cases (N = 1,330) were diagnosis of breast cancer at or before 45 years and self-reported race/ethnicity plus grandparents’ country of origin information consistent with Caucasian, East Asian, Hispanic/Latino, or African American racial/ethnic heritage. The controls (N = 1,123) were frequency matched to the cases within each center on racial/ethnic group, with age at selection not more than ±10 years from the age at diagnosis range of the patients gathered from the same center. Because of the shortage of available controls in some ethnic/ethnic and age groups, the frequency matching was not one-to-one in all subgroups (Table 4). According to the BCFR records, 25.4% of the selected cases had at least one first-degree relative with invasive breast cancer (*vs* 9.8% of the controls), 35.7% of the cases had at least one second-degree relative with invasive breast cancer (*vs* 21.6% of the controls), 3.4% of the cases had at least one first-degree relative with ovarian cancer (*vs* 2.3% of the controls), and 4.8% of the cases had at least one second-degree relative with ovarian cancer (*vs* 4.1% of the controls).

**Table 4:** Distribution of patients and controls by study center and by race/ethnicity in the BCFR.

Study Center	Race/Ethnicity	Cases	Controls	*Total*
Australian BCFR				
	Caucasian	560	510	*1,070*
	East Asian	28	13	*41*
	Latina	8	1	*9*
Ontario BCFR				
	Caucasian	301	459	*760*
	East Asian	8	4	*12*
	Latina	4	0	*4*
Northern California	East Asian	177	54	*231*
BCFR	Latina	146	46	*192*
	African American	98	36	*134*
	*Total*	*1,330*	*1,123*	*2,453*

In the analysis of the sequence variants, we excluded 5 subjects due to a PCR failure rate higher than 20% of the coding sequence. These 5 subjects correspond to 3 Australian controls, 1 Canadian control and 1 Canadian case; none of them carried a rare variant in the part of the coding sequence that has been screened.

### Mutation Screening

Mutation screening started from 30ng of whole-genome amplified (WGA) DNA for coding exons 1-10. The laboratory process was as described in detail for our recent studies of *ATM* [7] and *CHEK2* [11]. Our semi-automated approach, tracked by a Laboratory Information Management System (LIMS) [50], [51], relies on mutation scanning by high-resolution melt curve (HRM) analysis followed by direct Sanger sequencing of the individual samples for which an aberrant melting curve profile is indicative of the presence of a sequence variant. In a previous work, we showed that the HRM technique showed high sensitivity and specificity (1.0, and 0.8, respectively, for amplicons of <400 bp) for mutation screening by comparing the results with those obtained with Sanger sequencing [52].

In brief, we designed nested PCR amplicons to screen the 10 coding exons of the gene. Primary PCR (PCR1), usually set up as a three amplicon triplex, was performed in an 8 µl reaction volume containing 30 ng of template DNA. Simplex secondary PCRs (PCR2) were then performed in 6 µl reaction volume containing 1.5 µl of 1∶100 diluted PCR1 product. Eight PCR1 (with amplicon ranging from 284 bp to 787 bp) and eleven PCR2 (with amplicon ranging from 236 bp to 336 bp) were designed to screening the entire coding sequence of the gene.

For *RAD51* amplicons harboring a SNP(s) with frequency ≥1% in either dbSNP or initial amplicon testing, we applied a simultaneous mutation scanning and genotyping approach using HRM analysis to improve the sensitivity and the efficiency of the mutation screening, as described previously [50], [51].

All exonic sequence variants, plus splice junction consensus sequence variants that reduced splice junction sequence similarity to the standard consensus sequences AG∧GTRRGT (donor) or Y_16_NYAG∧ (acceptor) (where ∧ indicates the position of the splice junction), were re-amplified from genomic DNA or from a second WGA reaction product for confirmation of the presence of the variant.

All samples that failed either at the primary PCR, secondary PCR, or sequencing reaction stage were re-amplified from WGA DNAs or genomic DNAs. Samples that still did not provide satisfactory mutation screening results for at least 80% of the *RAD51* coding sequence were excluded from the study (*N* = 5). Primer and probe sequences are available from the authors upon request.

### Genotyping of the 5′UTR −135G>C SNP (rs1801320)

A Taqman **(**Applied Biosystems, Warrington, UK) 5′-allele discrimination assay was used to genotype the RAD51 −135G/C SNP using the primer sequences GCAGCGCTCCTCTCTCCAGC (Forward 5′-3′) and CTGGGAACTGCAACTCATCT (reverse 5′-3′), and the Taqman minor groove binder (MGB) probe sequences 5′-CAACGCCCGTGGCTTACGCT-3′ and CCCCAACGCCCCTGGCTTAC-3′. The probes were labeled with the fluorescent dyes FAM and VIC, respectively. The PCR reaction was performed in a total volume of 5 µl with the following amplification protocol: denaturation at 95°C for 15 min, followed by 40 cycles of denaturation at 95°C for 15 seconds and annealing and extension at 60°C for 1 minute. After PCR, the genotype of each sample was attributed automatically by measuring the allele-specific fluorescence with ABI Prism 7900 HT Sequence Detection System using the SDS 2.1 software for allelic discrimination (Applied Biosystems).

### Alignments and Scoring of Missense Substitutions

We used M-coffee (http://tcoffee.crg.cat/apps/tcoffee/do:mcoffee), which is part of the T-Coffee (Tree-based Consistency Objective Function for alignment Evaluation) software suite of alignment tools [53], [54], to prepare a RAD51 protein multiple sequence alignment with 15 RAD51 orthologs, in which the most diverged sequence was from fruitfly (Drosophila melanogaster), to predict the effect of the missense substitutions identified in *RAD51* on the gene product. The alignment was characterized by (1) determining percentage sequence identity between each pair of sequences in the alignment, (2) using the Protpars routine of Phylogeny Inference Package version 3.2 software (PHYLIP) [55] to make a maximum parsimony estimate of the number of substitutions that occurred along each clade of the underlying phylogeny and (3) recording the “median sequence conservation score” reported by the missense substitution analysis program Sorting Intolerant from Tolerant (SIFT) [56]. The sequence alignment. or updated versions thereof, is available at the Align-GVGD website [http://agvgd.iarc.fr/]). Missense substitutions observed during our mutation screening of *RAD51* were scored using the Align-GVGD and SIFT software programs with our curated alignments and with PolyPhen2.1 software, using its precompiled alignment [57]. In brief, Align-GVGD grades variants in the query sequence based on a combination of Grantham Variation (*GV*), which measures the amount of observed biochemical evolutionary variation at a particular position in the alignment, and Grantham Deviation (*GD*), which measures the biochemical difference between the missense residue and the range of variation observed at its position in the protein. The classifier provides 7 ordered grades (C65, C55, C45, C35, C25, C15 and C0) ranging from the most likely deleterious to least likely deleterious [36]. SIFT “Sorts Intolerant From Tolerant” is a sequence homology-based tool that predicts variants in the query sequence as “neutral” or “deleterious” using normalized probabilities calculated from the input multiple sequence alignment [56]. Variants at a position with normalized probabilities less than 0.05 are predicted deleterious and predicted neutral with a probability greater than or equal to 0.05. PolyPhen-2 predicts variants as “benign,” “possibly damaging,” or “probably damaging” based on eight sequenced-based and three structure-based predictive features. The alignment pipeline used in PolyPhen-2 selects homologous sequences using a clustering algorithm and then constructs and refines the alignment yielding an alignment containing both orthologs and paralogs that may or may not be full length, which yields a wider breadth of sequences but decreased depth compared with the Align-GVGD alignment [57].

### *In silico* Prediction on Splicing

Missense and silent substitutions and intronic variants detected in the vicinity of splice junction consensus sites on splicing with NNSplice [37] and MaxEntScan [38] programs. NNSplice employs separate feedforward neural networks with one layer of hidden units to recognize acceptor and donor sites. Positions other than the original site and having more probability of being a splice site depending on the score from the neural network are predicted to create *de novo* splice site (*i.e.,* acceptor/donor). The output of the network is a score between 0 (low probability) and 1 (high probability) for a potential splice site. MaxEntScan’s algorithm computes the maximum entropy score of a given sequence using human training splice site models. It is used for scoring the fitness of potential splice donor or splice acceptor sequences. If the MES acceptor or donor score is close or higher that the score obtained for native acceptor or donor splicing site, the variant is likely to create a *de novo* the splice junction.

### Statistical Analysis

Analyses were performed using the chi square test and multivariable unconditional logistic regression using Stata version 11 software (StataCorp, College Station, TX, USA). Differences in the case-control ratio between racial/ethnic groups and age categories were accounted for by including categorical variables for each age category and racial/ethnic group. Adjustment was also made for study center. Adjustment for racial/ethnic group should also capture confounding of genetic and social factors with interaction terms, allowing that this confounding effect may be different for the broadly labeled racial/ethnic groups in different centers. Because the BCFR matched cases and controls on age in 5-year categories, and because the maximum age of BCFR patients included in this study was 45 years, all participants ages 41 years and older (at diagnosis for patients and at ascertainment for controls) were combined into a single age category.

## Supporting Information

Table S1 Stratified analyses of *RAD51* non-coding SNPs on breast cancer risk in the BCFR.
(DOCX)
