# Supplementary material for: RAD51 and Breast Cancer Susceptibility: No Evidence for Rare Variant Association in the Breast Cancer Family Registry Study
Source: PLoS One. 2012 Dec 27;7(12):e52374. doi: 10.1371/journal.pone.0052374 (PMC3531476; doi:10.1371/journal.pone.0052374)
Supplement: Table S1 — Stratified analyses of RAD51 non-coding SNPs on breast cancer risk in the BCFR. (DOCX) [file pone.0052374.s001.docx]

**Supplementary Table S1. Stratified analyses of *RAD51* non-coding SNPs on breast cancer risk in the BCFR.**

| SNP | Population | Number of genotyped subjects | Minor allele frequency |  | Log-additive model^b^ | |
| --- | --- | --- | --- | --- | --- | --- |
|  |  | Cases / Controls | Cases / Controls | Chi^2^ *P*-value^a^ | OR [95% CI] | *P*-trend |
| 5’UTR | Combined | 1,193 / 1,019 | 0.089 / 0.080 | 0.42 | 0.89 [0.71, 1.27] | 0.30 |
|  | By race/ethnicity |  |  |  |  |  |
|  | European | 763 / 873 | 0.061 / 0.066 | 0.56 | 0.95 [0.71, 1.27] | 0.71 |
|  | East Asian | 187 / 69 | 0.131 / 0.203 | **0.044** | **0.56 [0.33, 0.95]** | **0.033** |
|  | Recent African ancestry | 91 / 32 | 0.236 / 0.203 | 0.59 | 1.36 [0.69, 2.71] | 0.38 |
|  | Latina | 152 / 45 | 0.072 / 0.078 | 0.86 | 0.87 [0.33, 2.29] | 0.77 |
| c.226-72delA | Combined | 1,249/ 1,063 | 0.073 / 0.064 | 0.26 | 1.01 [0.79, 1.30] | 0.94 |
|  | By race/ethnicity |  |  |  |  |  |
|  | European | 799 / 911 | 0.059 / 0.055 | 0.67 | 1.13 [0.83, 1.53] | 0.44 |
|  | East Asian | 194 / 69 | 0.116 / 0.167 | 0.13 | 0.64 [0.37, 1.11] | 0.11 |
|  | Recent African ancestry | 98 / 36 | 0.117/ 0.097 | 0.64 | 1.38 [0.54, 3.55] | 0.50 |
|  | Latina | 158 / 47 | 0.063 / 0.064 | 0.99 | 0.98 [0.35, 2.70] | 0.96 |
| c.226-70T>A | Combined | 1,254 / 1,066 | 0.291 / 0.341 | **0.0003** | 1.00 [0.87, 1.15] | 0.95 |
|  | By race/ethnicity |  |  |  |  |  |
|  | European | 804 / 913 | 0.366 / 0.373 | 0.66 | 1.00 [0.86, 1.16] | 0.95 |
|  | East Asian | 194 / 70 | 0.108 / 0.093 | 0.61 | 1.29 [0.66, 2.54] | 0.46 |
|  | Recent African ancestry | 98 / 36 | 0.107 / 0.153 | 0.31 | 0.58 [0.24, 1.43] | 0.24 |
|  | Latina | 158 / 47 | 0.247 / 0.223 | 0.64 | 1.21 [0.69, 2.14] | 0.51 |
| c.226-33T>G | Combined | 1,255 / 1,066 | 0.266 / 0.206 | **<0.0001** | 0.95 [0.81, 1.10] | 0.48 |
|  | By race/ethnicity |  |  |  |  |  |
|  | European | 805 / 913 | 0.157/ 0.168 | 0.36 | 0.86 [0.71, 1.04] | 0.12 |
|  | East Asian | 194 / 70 | 0.639 / 0.579 | 0.20 | 1.25 [0.85, 1.83] | 0.26 |
|  | Recent African ancestry | 98 / 36 | 0.214 / 0.208 | 0.92 | 1.04 [0.51, 2.13] | 0.91 |
|  | Latina | 158 / 47 | 0.399 / 0.383 | 0.78 | 1.06 [0.66, 1.68] | 0.82 |
| c.344-36T>G | Combined | 1,255 / 1,066 | 0.074 / 0.073 | 0.75 | 0.92 [0.72, 1.17] | 0.51 |
|  | By race/ethnicity |  |  |  |  |  |
|  | European | 805 / 913 | 0.063/ 0.062 | 0.86 | 1.05 [0.79, 1.40] | 0.75 |
|  | East Asian | 194 / 70 | **0.106/ 0.186** | **0.015** | **0.50 [0.28, 0.87]** | **0.015** |
|  | Recent African ancestry | 98 / 36 | 0.214 / 0.208 | 0.64 | 1.38 [0.54, 3.55] | 0.50 |
|  | Latina | 158 / 47 | 0.060 / 0.064 | 0.90 | 0.95 [0.34, 2.64] | 0.92 |
| c.531-48T>G | Combined | 1,253 / 1,063 | 0.006/ 0.002 | 0.06 | 1.45 [0.48, 4.39] | 0.51 |
|  | By race/ethnicity |  |  |  |  |  |
|  | European | 803 / 910 | 0 / 0.001 | 0.50 | NA | NA |
|  | East Asian | 194 / 70 | 0 / 0 | NA | NA | NA |
|  | Recent African ancestry | 98 / 36 | 0.077/ 0.042 | 0.42* | 2.43 [0.64, 9.19] | 0.19 |
|  | Latina | 158 / 47 | 0 / 0 | NA | NA | NA |
| c.644+29T>A | Combined | 1,253 / 1,063 | 0.011 / 0.002 | **0.0002** | 2.83 [0.91, 8.78] | 0.072 |
|  | By race/ethnicity |  |  |  |  |  |
|  | European | 803 / 910 | 0 / 0 | NA | NA | NA |
|  | East Asian | 194 / 70 | 0 / 0 | NA | NA | NA |
|  | Recent African Ancestry | 98 / 36 | 0.122 / 0.042 | 0.07 | 3.48 [0.95, 12.76] | 0.06 |
|  | Latina | 158 / 47 | 0.001/ 0.011 | 1.00* | 0.78 [0.07, 8.39] | 0.84 |
| c.896+86C>T | Combined | 1,254 / 1,063 | 0.012 / 0.002 | **0.0001** | 3.01 [1.00, 9.10] | 0.051 |
|  | By race/ethnicity |  |  |  |  |  |
|  | European | 804 / 910 | 0 / 0 | NA | NA | NA |
|  | East Asian | 194 / 70 | 0 / 0 | NA | NA | NA |
|  | Recent African ancestry | 98 / 36 | **0.133 / 0.042** | **0.044** | **3.71 [1.04, 13.21]** | **0.043** |
|  | Latina | 158 / 47 | 0.010/ 0.011 | 1.00 | 0.78 [0.07, 8.39] | 0.84 |

^a^Test for the difference in C allele frequency between cases and controls.

^b^Results of the logistic regression assuming a log-additive model with study center and age included in the regression model as covariates in the combined analysis, and with race/ethnicity, study center and age as covariates in the stratified analysis.

*This result coresponds to FET *P*-value due to small number of observations
